# Supplementary material for: CRISPR/Cas9-Mediated Knockout of miR-130b Affects Mono- and Polyunsaturated Fatty Acid Content via PPARG-PGC1α Axis in Goat Mammary Epithelial Cells
Source: Int J Mol Sci. 2022 Mar 26;23(7):3640. doi: 10.3390/ijms23073640 (PMC8998713; doi:10.3390/ijms23073640)
Supplement: Supplementary file 1 [file ijms-23-03640-s001.zip › ijms-1619571-supplementary.pdf]

International Journal of
Molecular Sciences

MDPI

Article

CRISPR/Cas9-Mediated Knockout of miR-130b Affects Mono- and Polyunsaturated Fatty Acid Content via PPARG-PGC1α Axis in Goat Mammary Epithelial Cells

Lian Huang, Jun Luo *, Ning Song, Wenchang Gao, Lu Zhu and Weiwei Yao

Shaanxi Key Laboratory of Molecular Biology for Agriculture, College of Animal Science and Technology, Northwest A&F University, Yangling 712100, China; kin8248806@163.com (L.H.); songning@nwafu.edu.cn (N.S.); gaowenchang666@163.com (W.G.); 411143710@qq.com (L.Z.); 1971119605@qq.com (W.Y.)

* Correspondence: luojun@nwafu.edu.cn

Citation: Huang, L.; Luo, J.; Song, N.; Gao, W.; Zhu, L.; Yao, W. CRISPR/Cas9-Mediated Knockout of miR-130b Affects Mono- and Polyunsaturated Fatty Acid Content via PPARG-PGC1α Axis in Goat Mammary Epithelial Cells. *Int. J. Mol. Sci.* **2022**, *23*, 3640. https://doi.org/10.3390/ijms23073640

Academic Editor: Jan Wijnholds

Received: 15 February 2022
Accepted: 24 March 2022
Published: 26 March 2022

Publisher's Note: MDPI stays neutral with regard to jurisdictional claims in published maps and institutional affiliations.

Copyright: © 2022 by the authors. Licensee MDPI, Basel, Switzerland. This article is an open access article distributed under the terms and conditions of the Creative Commons Attribution (CC BY) license (https://creativecommons.org/licenses/by/4.0/).

Table S1. The siRNA Sequences of *PGC1α* and *PPARG*.

Name	Sense sequence (5´-3´)	Antisense sequence (3´-5´)
siNC	ACUACCGUUGUUAUAGGUGTT	CACCUAUAACAACGGUAGUTT
siPGC1α	GCCAACACUCAGCUAAGUUTT	AACUUAGCUGAGUGUUGGCTT
siPPARG-195	CCCGAUGGUUGCAGAUUAUTT	AUAAUCUGCAACCAUCGGGTT
siPPARG-980	GCGUGCACGAGAUCAUUUATT	UAAAUGAUCUCGUGCACGCTT
siPPARG-1164	CGGCUUAGCAAUAUUUAUATT	UAUAAAUAUUGCUAAGCCGTT

Table S2. Primers Used for sgRNA-induced 11 Off-Target Sites Amplification.

sgRNA	Off-target site	Primer sequence (5' to 3')	Size (bp)
sgRNA1	OT1	F: GGTGGTGGTTTCGGCTTGCTT<br>R: AACTGAGGTCTGCCTTCTTCCTTCC	771
	OT2	F: TCAGGGCTCCGTGCTAAAGAGG<br>R: TTCACCGAAGAGTTTGTGGACAGTAAAT	828
	OT3	F: CTCAACTATCTCGCCACTGCCCAACT<br>R: TGCCACGCAGCGTAGCCAAAA	592
	OT4	F: TGGCAAGTGGGACAAGAGGACA<br>R: CACATGCGGCTATTTGGAAAGAAA	510
	OT5	F: GTGGCAAGAAGCCGAGGGCAAAC<br>R: CCGAGATCCCAACCAAACTATGACAGG	423
	OT6	F: CATGTGCGTGTATCTAATTTCCTATC<br>R: ATGACAACAGCAGCCATCAGTC	892
sgRNA2	OT1	F: ATCCCGTCTGCCTGTCACCCATCC<br>R: CCGACTTCTCCTCAGTCCGGCTTCAC	897
	OT2	F: GGGAAAGATTGAGGATGGGAGG<br>R: GCGAAGTTGCTGTATTTGCCGTAT	853
	OT3	F: AGCTGATGGCATGAATGGGTCG<br>R: CCTGACGAGGAGGTTCACCAGAAA	935
	OT4	F: CCCTTATCTTCTGTGGCATAAACTCC<br>R: TTCTGCTACAAACCCGACTCCC	703
	OT5	F: GCGGGCTGTAGTCCAAGAAG<br>R: AGCTGAGCGCCGAAGAATT	716

Table S3. Primers for RT-qPCR of miRNAs by S-Poly(T) Plus Method.

miRNA	Primer sequence (5' to 3')
miR-130b-3p	Reverse:<br>GTGCAGGGTCCGAGGTCAGAGCCACCTGGGCAATTTTTTTTTTT<br>ATGCCC<br>Forward: TTCGGCAGTGCAATGATGAAA
miR-130b-5p	Reverse:<br>GTGCAGGGTCCGAGGTCAGAGCCACCTGGGCAATTTTTTTTTTT<br>AGTAGT<br>Forward: CGGACTCTTTCCCTGTTGC
miR-130a-3p	Reverse:<br>GTGCAGGGTCCGAGGTCAGAGCCACCTGGGCAATTTTTTTTTTT<br>GCCCTT<br>Forward: TGTCGGCAGTGCAATGTTAAA
miR-301b-3p	Reverse:<br>GTGCAGGGTCCGAGGTCAGAGCCACCTGGGCAATTTTTTTTTTT<br>GCTTTG<br>Forward: TCGGCAGTGCAATGATATTGT
18S rRNA	Reverse:<br>GTGCAGGGTCCGAGGTCAGAGCCACCTGGGCAATGCAGTGATG<br>GCAAAGG<br>Forward: CAGCACATCTTGCGAGTACTC

Universal reverse primer	CAGTGCAGGGTCCGAGGT

Table S4. Characteristics of Primers for RT-qPCR of mRNA.

NCBI accession no.	Gene	Primer sequence (5’ to 3’)	Size (bp)
JN236219.1	*ACACA*	F: CTCCAACCTCAACCACTACGG<br>R: GGGGAATCACAGAAGCAGCC	171
NM_001035289.2	*ACOX1*	F: CGAGTTCATTCTCAACAGTCCT<br>R: GCATCTTCAAGTAGCCATTATCC	211
BC119914	*ACSL1*	F: GTGGGCTCCTTTGAAGAACTGT<br>F: ATAGATGCCTTTGACCTGTTCAAAT	120
BC134532	*ACSS2*	F: GGCGAATGCCTCTACTGCTT<br>R: GGCCAATCTTTTCTCTAATCTGCTT	100
HQ846827	*ADFP*	F: TACGATGATACAGATGAATCCCAC<br>R: CAGCATTGCGAAGCACAGAGT	202
NM_00108366.1	*AGPAT6*	F: AAGCAAGTTGCCCATCCTCA<br>R: AAACTGTGGCTCCAATTTCGA	101
NM_001285739.1	*ATGL*	F: GGAGCTTATCCAGGCCAATG<br>R: TGCGGGCAGATGTCACTCT	180
X91503	*CD36*	F: GTACAGATGCAGCCTCATTTCC<br>R: TGGACCTGCAAATATCAGAGGA	81
FJ415874.1	*CPT1A*	F: AAGGACCTCTACGCCAACACG<br>R: TTTGCGGTGGACGATGGAG	267
DQ380249.1	*DGAT1*	F: CCACTGGGACCTGAGGTGTC<br>R: GCATCACCACACACCAATTCA	101
NM_001046597.1	*ELOVL5*	F: GTTCTGTGAGTTAGTGACGGGAGT<br>R: GTGGTTGTTCTTGCGGAGGAT	175
NM_001046597.1	*ELOVL6*	F: GGAAGCCTTTAGTGCTCTGGTC<br>R: ATTGTATCTCCTAGTTCGGGTGC	205
NM_174313.2	*FABP3*	F: GATGAGACCACGGCAGATG<br>R: GTCAACTATTTCCCGCACAAG	120
EE347846	*FADS1*	F: GGTGGACTTGGCCTGGATG<br>R: TGACCATGAAGACAAGCCCC	101
DV895683	*FADS2*	F: GCCTGTAGGCTCAGATGTTTGTTC<br>R: TGCCTGGCAGTAACAGAGCAC	101
DQ915966.3	*FASN*	F: GGGCTCCACCACCGTGTTCCA<br>R: GCTCTGCTGGGCCTGCAGCTG	226
AY515690	*GPAM*	F: ATTGACCCTTGGCACGATAG<br>R: AACAGCACCTTCCCACAAAG	188
EU273879	*HSL*	F: GGGAGCACTACAAACGCAACG<br>R: TGAATGATCCGCTCAAACTCG	118
NM_001286088.1	*INSIG1*	F: AGCCTCACAAGTTCAAGCG<br>R: ACAGTGCTGCTAATGTCAAGG	132
NM_001285738	*INSIG2*	F: ATACCCCTGCATTGACAGACAT<br>R: ACCAAGGCCAAAACCACTTC	109
GU332719	*LXRA*	F: CATCAACCCCATCTTCGAGTT<br>R: CAGGGCCTCCACATATGTGT	163
XM_005681211.1	*PPARA*	F: CGGTGTCCACGCATGTGA<br>R: TCAGCCGAATCGTTCTCCTAAA	56
HQ589347.1	*PPARG*	F: CCTTCACCACCGTTGACTTCT<br>R: GATACAGGCTCCACTTTGATTGC	145
DT860044	*RPS9*	F: CCTCGACCAAGAGCTGAAG<br>R: CCTCCAGACCTCACGTTTGTTC	64
GU947654	*SCD1*	F: CCATCGCCTGTGGAGTCAC<br>R: GTCGGATAAATCTAGCGTAGCA	257
HM443643.1	*SREBP1a*	F: CTGCTGACCGACATAGAAGACAT<br>R: GTAGGGCGGGTCAAACAGG	81
HM443643.1	*SREBP1c*	F: CTGCTGACCGACATAGAAGACAT	81

		R: GTAGGGCGGGTCAAACAGG	
NC_030812.1	*SREBP2*	F: CGGCGTGATCGTGCTGAGCGTC<br>R: CCGACAGGCAGGTTTGCAGGTTGG	173
HQ846826	*TIP47*	F: GGTGGAGGGTCAGGAGAAA<br>R: TCACGGAACATGGCGAGT	123
NM_001037471	*UXT*	F: CAGCTGGCCAAATACCTTCAA<br>R: GTGTCTGGGACCACTGTGTCAA	125
BC102076	*XDH*	F: GATCATCCACTTTTCTGCCAATG<br>R: CCTCGTCTTGGTGCTTCCAA	100

ACACA, acetyl-coenzyme A carboxylase alpha; *ACOX1*, Acyl coenzyme A oxidase 1; *ACSL1*, acyl-CoA synthetase long-chain family member 1; *ACSS2*, Acyl-CoA Synthetase short-chain family member 2; *ADFP*, Adipose differentiation-related protein; *AGPAT6*, 1-acylglycerol-3-phosphate O-acyltransferase 6; *ATGL*, Adipose triglyceride lipase; *CD36*, Thrombospondin receptor; *C/EBPβ*, CCAAT enhancer binding protein beta; *CPT1A*, Carnitine palmitoyltransferase 1A; *DGAT1*, diacylglycerol acyl transferase 1; *DGAT2*, diacylglycerol acyl transferase 2; *ELOVL6*, elongase of very long chain fatty acids 6; *FADS1*, fatty acid delta-6-desaturase 1; *FADS2*, fatty acid delta-6-desaturase 2; *FASN*, fatty acid synthase; *GPAM*, Glycerol-3-phosphate acyltransferase; *HSL*, Hormone-sensitive lipase; *INSIG1*, Insulin induced protein 1; *INSIG2*, Insulin induced protein 2; *PPARA*, peroxisome proliferator-activated receptor *α*; *RPS9*, ribosomal protein S9; *SCAP*, SREBP cleavage activating protein; *SCD1*, stearoyl-CoA desaturase 1; *SREBP1a*, Sterol regulatory element-binding transcription protein 1a; *SREBP1c*, Sterol regulatory element-binding transcription protein 1c; *SREBP2*, Sterol regulatory element-binding transcription protein 2; *TIP47*, Tail-interacting protein 47; *UXT*, ubiquitously expressed transcript; *XDH*, Xanthine dehydrogenase.

Table S5. Effects of *PGC1α* Silencing on Fatty Acid Composition in miR-130b Knockout GMEC.

Fatty acid (%)	Control+siNC	Control+siPGC1α	miR-130b KO+siNC	miR-130b KO+siPGC1α
C14:0	3.72±0.10[a]	3.00±0.20[b]	2.98±0.10[b]	2.78±0.21[b]
C15:0	0.77±0.02[a]	0.78±0.05[a]	0.70±0.00[b]	0.73±0.03[ab]
C16:0	48.36±0.47[a]	48.01±0.32[ab]	47.47±0.31[b]	47.83±0.08[ab]
C16:1	1.92±0.03[a]	1.98±0.08[a]	1.57±0.02[c]	1.67±0.02[b]
C17:0	1.20±0.01[c]	1.31±0.02[a]	1.25±0.03[b]	1.33±0.03[a]
C17:1	0.47±0.01[a]	0.46±0.01[ab]	0.43±0.01[b]	0.44±0.02[ab]
C18:0	23.68±0.31[b]	24.30±0.66[b]	25.69±0.16[a]	25.11±0.13[a]
C18:1	13.23±0.17[ab]	13.39±0.13[a]	13.19±0.17[ab]	12.98±0.24[b]
C18:2	1.78±0.01[ab]	1.80±0.05[a]	1.72±0.05[b]	1.81±0.03[a]
C20:0	0.30±0.02[b]	0.30±0.02[b]	0.34±0.01[a]	0.32±0.01[ab]
C20:3	0.65±0.00[b]	0.64±0.01[b]	0.76±0.01[a]	0.77±0.02[a]
C20:4	2.47±0.10	2.47±0.12	2.43±0.09	2.62±0.03
C20:5	0.80±0.02[b]	0.98±0.07[a]	0.77±0.02[b]	0.93±0.02[a]
C22:6	0.65±0.01[b]	0.59±0.02[c]	0.69±0.00[a]	0.67±0.02[ab]
SFA*	78.03	77.69	78.44	78.10
MUFA**	15.62	15.83	15.19	15.10
PUFA***	6.35	6.48	6.37	6.80

Statistical significance is as follows: lower case letters, $P < 0.05$. Data are presented as mean ± SEM.

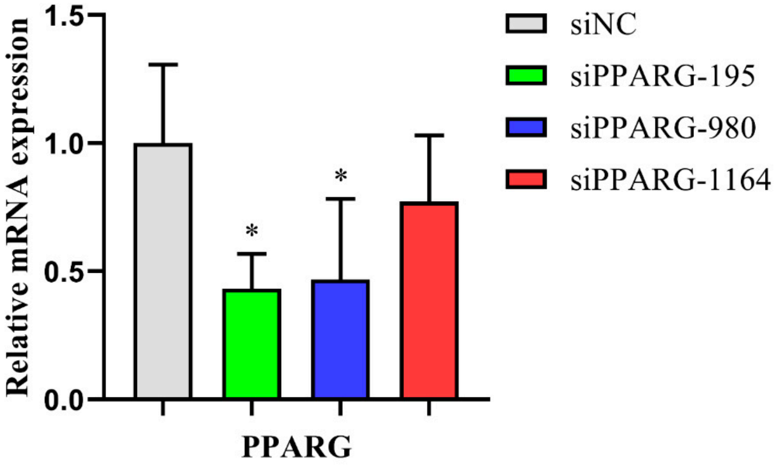

Figure S1. Verification of interference efficiency of siPPARG.
